# Overexpression of wheat ferritin gene *TaFER-5B* enhances tolerance to heat stress and other abiotic stresses associated with the ROS scavenging

**DOI:** 10.1186/s12870-016-0958-2

**Published:** 2017-01-14

**Authors:** Xinshan Zang, Xiaoli Geng, Fei Wang, Zhenshan Liu, Liyuan Zhang, Yue Zhao, Xuejun Tian, Zhongfu Ni, Yingyin Yao, Mingming Xin, Zhaorong Hu, Qixin Sun, Huiru Peng

**Affiliations:** State Key Laboratory for Agrobiotechnology, Key Laboratory of Crop Heterosis and Utilization (MOE), Beijing Key Laboratory of Crop Genetic Improvement, China Agricultural University, NO.2 Yuanmingyuan Xi Road, Haidian District, Beijing, 100193 China

**Keywords:** *TaFER-5B*, Heat stress, Abiotic stress, Ferritin-lacking mutant, Wheat, *Arabidopsis*

## Abstract

**Background:**

The yield of wheat (*Triticum aestivum* L.), an important crop, is adversely affected by heat stress in many regions of the world. However, the molecular mechanisms underlying thermotolerance are largely unknown.

**Results:**

A novel ferritin gene, *TaFER*, was identified from our previous heat stress-responsive transcriptome analysis of a heat-tolerant wheat cultivar (TAM107). *TaFER* was mapped to chromosome 5B and named *TaFER-5B*. Expression pattern analysis revealed that *TaFER-5B* was induced by heat, polyethylene glycol (PEG), H_2_O_2_ and Fe-ethylenediaminedi(o-hydroxyphenylacetic) acid (Fe-EDDHA). To confirm the function of *TaFER-5B* in wheat, *TaFER-5B* was transformed into the wheat cultivar Jimai5265 (JM5265), and the transgenic plants exhibited enhanced thermotolerance. To examine whether the function of ferritin from mono- and dico-species is conserved, *TaFER-5B* was transformed into *Arabidopsis*, and overexpression of *TaFER-5B* functionally complemented the heat stress-sensitive phenotype of a ferritin-lacking mutant of *Arabidopsis*. Moreover, *TaFER-5B* is essential for protecting cells against heat stress associated with protecting cells against ROS. In addition, *TaFER-5B* overexpression also enhanced drought, oxidative and excess iron stress tolerance associated with the ROS scavenging. Finally, *TaFER-5B* transgenic *Arabidopsis* and wheat plants exhibited improved leaf iron content.

**Conclusions:**

Our results suggest that *TaFER-5B* plays an important role in enhancing tolerance to heat stress and other abiotic stresses associated with the ROS scavenging.

**Electronic supplementary material:**

The online version of this article (doi:10.1186/s12870-016-0958-2) contains supplementary material, which is available to authorized users.

## Background

Most of the world’s wheat growing areas are frequently subject to heat stress during the growing season. High temperatures adversely affect wheat yield and quality [1]. Over the past three decades (1980–2008), heat stress has caused a decrease of 5.5% in global wheat yields [2]. Thus, research on the molecular mechanism of thermotolerance and the development of new wheat tolerant varieties using classical breeding techniques and biotechnological approaches is increasingly important. As sessile organisms, plants have evolved various response mechanisms to adapt to abiotic stress, particularly molecular responses to maintain normal life activities [3–6]. Genes that respond to adverse growth conditions are essential for enhancing abiotic stress tolerance and developing stress-tolerant crops.

Iron is an essential nutrient for all cells. However, excess free iron is harmful to cells because it promotes the formation of free radicals via the Fenton reaction. Thus, iron homeostasis must be well controlled. As iron-storage proteins, ferritins play important roles in sequestering or releasing iron upon demand [7]. Ferritins are a class of 450-kDa proteins consisting of 24 subunits, which are present in all cell types [8]. In contrast to animal ferritins, subcellular localization of plant ferritins in the cytoplasm has not been reported. Plant ferritins are exclusively targeted to plastids and mitochondria [9–12]. The model plant *Arabidopsis* contains four ferritin genes: *AtFER1*, *AtFER2*, *AtFER3* and *AtFER4*. Hexaploid wheat contains two ferritin genes that map to chromosomes 5 and 4, and each of the individual homeoalleles can be located to the A, B or D genome [13]. Thus, ferritin genes are conserved throughout the plant kingdom, and two genes per genome have been identified in all studied cereals [13].

Transcriptome analysis of plant responses to stress has identified a number of genes. In plants, ferritin gene expression was induced in response to drought, salt, cold, heat and pathogen infection [9, 14]. *Arabidopsis* ferritin genes were induced by treatment with H_2_O_2_, iron and abscisic acid (ABA); however, not all four *AtFER* genes were induced [15]. Ferritin was up-regulated in response to drought in the SSH (Suppression Subtractive Hybridization) cDNA library of soybean nodules [16]. Overexpression of ferritin also significantly improved abiotic stress tolerance in grapevine plants [17].

Oxidative damage of biomolecules is a common trait of abiotic stress. If oxidative damage is not well controlled, it can ultimately trigger programmed cell death (PCD) [18]. Thus, reactive oxygen species (ROS) must be tightly managed by enhancing ROS scavenging and/or reinforcing pathways preventing ROS production. In addition to buffering iron, previous studies have also revealed that plant ferritins protect cells against oxidative damage [19]. However, little information is known about ferritin gene functions involved in tolerance to heat and other abiotic stresses. We previously analysed the genome-wide expression profiles of wheat under heat-stress conditions and identified a large number of genes responding to heat stress, including ferritin genes [14]. In the present study, the expression patterns of *TaFER-5B* in seedlings treated by various stress were studied, and the relationship between ferritins and thermotolerance was elaborated.

## Results

### Cloning of a ferritin-encoding gene, *TaFER-5B*, from wheat (*Triticum aestivum* L.)

**Fig. 1:**
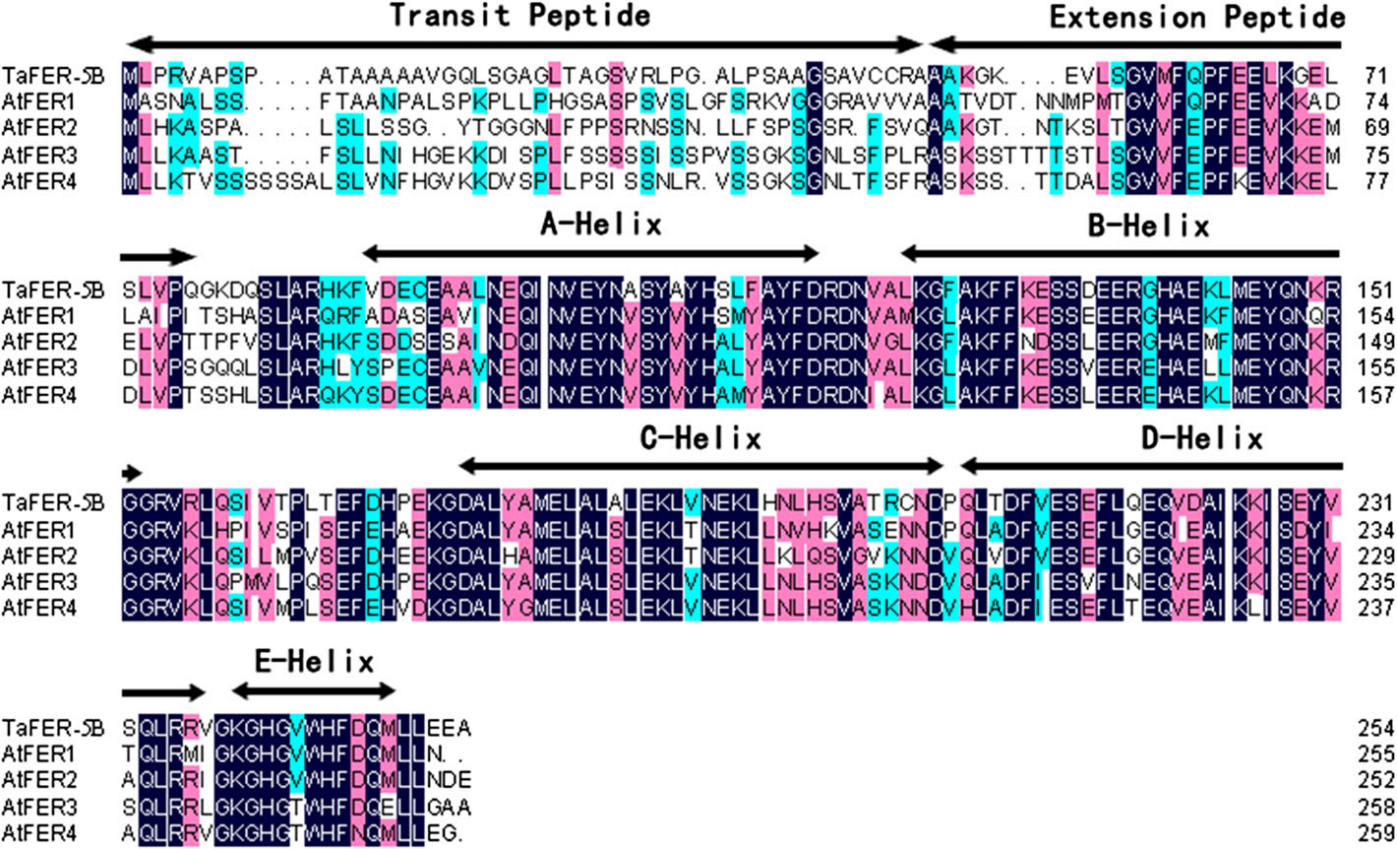
Sequence alignment of *TaFER-5B* and previously reported ferritin genes *AtFER1-4* in *Arabidopsis*. Regions corresponding to the ferritin domains are indicated by arrows

Microarray analysis using the Affymetrix Genechip® Wheat Genome Array indicated that the probe Ta.681.1.S1_x_at was induced 2^9.08^-fold after high-temperature treatment for 1 h [14]. Based on the probe sequence, we cloned the full-length open reading frame of this gene (*TaFER*, accession no. GenBank KX025176; Additional file 1) from wheat cultivar “TAM107”. The coding sequence shares 97.15%, 99.66%, and 97.15% homology with sequences on chromosomes 5A, 5B, and 5D, respectively, of the recently published wheat cultivar Chinese Spring (CS) genome (International Wheat Genome Sequencing Consortium, 2014). The sequence on chromosome 5B corresponds to the original heat-responsive transcript named *TaFER-5B*. Comparison of the amino acid sequences between *TaFER-5B* and ferritin genes from the model plant *Arabidopsis* revealed that *TaFER-5B* is a conserved gene containing the transit peptide domain responsible for plastid localization, an adjoined extension peptide domain involved in protein stability and five helixes (Fig. 1) [20–22]. The amino acid sequence of *TaFER-5B* exhibits 60.47% identity with *AtFER1*, 62.26% identity with *AtFER2*, 61.69% identity with *AtFER3*, and 60.92% identity with *AtFER4* (Fig. 1), and phylogenetic tree analysis revealed higher identity with the ferritins from maize, rice and barley (Additional file 2: Figure S1; Additional file 3).

### The *TaFER-5B* gene is expressed in response to different stress treatments

**Fig. 2:**
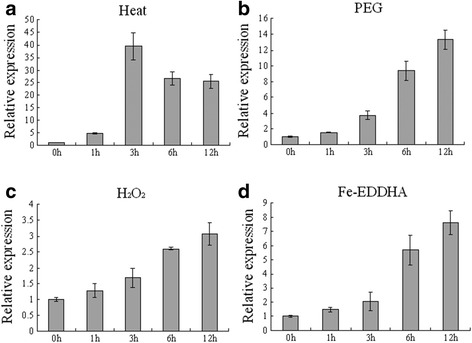
Expression pattern of *TaFER-5B* under heat (**a**), PEG (**b**), H_2_O_2_ (**c**) and Fe-EDDHA treatment (**d**) as assessed by RT-qPCR. The data represent the means of three replicates ± SD

The expression patterns of ferritin genes in wheat under diverse abiotic stress conditions were analysed by RT-qPCR using gene-specific primers (Fig. 2). *TaFER-5B* expression increased significantly and peaked at 3 h after heat treatment at 40 °C. After long-term heat-stress treatment (12 h), *TaFER-5B* expression decreased, but increased mRNA abundance was maintained (Fig. 2a). We also analysed the expression level of *TaFER-5B* under PEG, H_2_O_2_ and Fe-EDDHA conditions. *TaFER-5B* expression gradually increased and peaked at 12 h of treatment (Fig. 2b, c and d). These results demonstrate that *TaFER-5B* expression is induced by heat, PEG, H_2_O_2_ and Fe-EDDHA treatment.

### *TaFER-5B* overexpression in wheat enhances thermotolerance at the seedling stage

To gain insight into the function of *TaFER-5B*, *TaFER-5B* under the control of the maize ubiquitin promoter was transformed into wheat cultivar Jimai5265 (JM5265) by particle bombardment. In total, 15 transgenic events were produced, and integration of the ferritin gene was confirmed by PCR analysis with specific corresponding primers. The transgenic lines were analysed over the T_1_ and T_2_ generations. Three lines (W-L1, W-L2 and W-L3) that exhibited up-regulation of *TaFER-5B* in shoots at the early seedling stage (Additional file 4: Figure S2) were selected for further analysis.

**Fig. 3:**
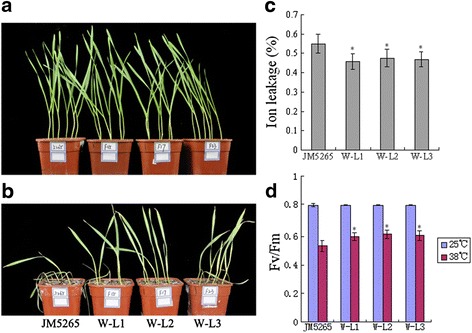
Thermotolerance assay of *TaFER-5B* transgenic wheat plants at the seedling stage. **a** Phenotype of 10-day-old JM5265 and three *TaFER-5B* transgenic wheat lines, W-L1, W-L2 and W-L3, before heat treatment. **b** 5-day-old JM5265 and three *TaFER-5B* transgenic wheat lines, W-L1, W-L2 and W-L3, were treated at 45 °C for 18 h, and photographs were taken after 5 d recovery at 22 °C. **c** Ion leakage assay of the seedlings in (**a**) after heat treatment. **d** Maximum efficiency of PSII photochemistry (Fv/Fm ratio) in the seedlings in (**a**) after heat treatment at 38 °C for 2 h. The data represent mean values ± SD of three independent experiments. (* indicates significance at *P* < 0.05)

Growth and stress resistance phenotypes were investigated at the seedling stage grown under normal and heat-stress conditions. Under normal conditions, the *TaFER-5B* transgenic lines exhibited no obvious differences (Fig. 3a). However, under heat-stress conditions, wild type (WT) wilted more rapidly than the *TaFER-5B* transgenic lines after heat stress at 45 °C for 18 h and recovery at 22 °C for 5 d. (Fig. 3b). As a parameter for evaluating stress-induced membrane injury, electrolyte leakage is often used to analyse plant tolerance to stress. Thus, we further evaluated electrolyte leakage with detached leaves under heat-stress conditions. The *TaFER-5B* transgenic lines exhibited reduced electrolyte leakage with detached leaves compared with JM5265 under heat-stress conditions (Fig. 3c). Under heat-stress conditions, photosynthetic activity was markedly reduced and accompanied by direct and indirect photosynthetic system damage. The ratio of variable to maximal fluorescence (Fv/Fm) is an important parameter used to assess the physiological status of the photosynthetic apparatus. Environmental stress that affects photosystem II efficiency decreases Fv/Fm. Previous studies have indicated that disturbance of the electron flow under moderate heat stress might be an important determinant of heat-derived damage of the photosynthetic system [23]. Fv/Fm values in *TaFER-5B* transgenic lines were increased compared with JM5265 under heat-stress conditions, whereas no significant difference was observed under control conditions (Fig. 3d). These results indicate that *TaFER-5B* protects photosynthetic activity under heat-stress conditions.

### *Arabidopsis* ferritin-lacking mutants display heat stress-sensitive phenotypes and are rescued by *TaFER-5B* overexpression

The role of the ferritin gene in thermotolerance in *Arabidopsis* has not been characterized. To determine whether the function of ferritin from mono- and dico-species is conserved, the *Arabidopsis* ferritin gene triple mutant *fer1-3-4* (lacking three isoforms expressed in vegetative tissues, *AtFER1*, *3* and *4*) and quadruple mutant *fer1-2-3-4* (lacking all ferritin isoforms) were created by crossing the single mutant as described previously [19, 24] (Additional file 5: Figure S3). Then, we transformed *35S::TaFER-5B* into WT and *fer1-2-3-4* plants. Detection of protein expression levels by Western blot analysis revealed that the expression level of ferritin increased in the overexpression lines (A-L1 and A-L2) compared with WT (Additional file 6: Figure S4A) but decreased in *fer1-2-3-4* and *fer1-3-4* plants compared with WT (Additional file 6: Figure S4B). As shown in Additional file 6: Figure S4B, ferritin protein levels in *fer1-2-3-4* plants complemented by the *TaFER-5B* gene (A-CL1) were very similar to those in WT plants. No obvious morphological differences were observed in the transgenic lines at different developmental stages (data not shown).

**Fig. 4:**
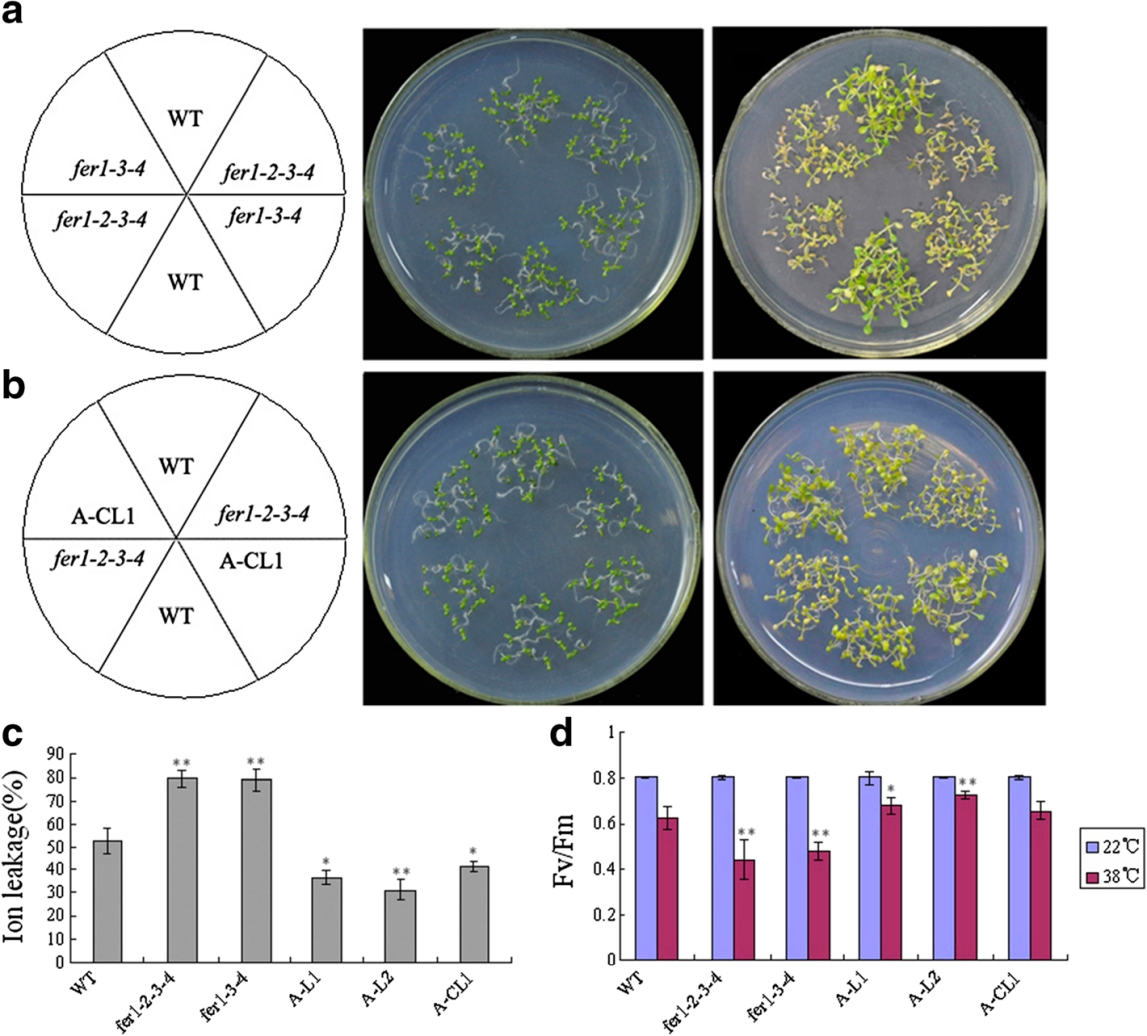
*Arabidopsis* ferritin-lacking mutants displaying a heat stress-sensitive phenotype were rescued by overexpression of *TaFER-5B*. **a** 6-day-old seedlings of WT, *fer1-3-4* and *fer1-2-3-4* were treated at 45 °C for 2 h, and photographs were taken after 7-d recovery at 22 °C. **b** 6-day-old seedlings of WT, *fer1-2-3-4* and A-CL1 (complemented line) were treated at 45 °C for 2 h, and photographs were taken after 7-d recovery at 22 °C. **c** Ion leakage assays of *fer1-2-3-4*, *fer1-3-4*, WT, overexpression lines A-L1 and A-L2 and complemented line A-CL1 seedlings after heat treatment. **d** Maximum efficiency of PSII photochemistry (Fv/Fm ratio) of *fer1-2-3-4*, *fer1-3-4*, WT, overexpression lines A-L1 and A-L2 and complemented line A-CL1 seedlings at 22 °C and 38 °C. The data represent mean values ± SD of three independent experiments. (* indicates significance at *P* < 0.05; ** indicates significance at *P* < 0.01)

Thus, *fer1-2-3-4, fer1-3-4, A-L1, A-L2, A-CL1* and WT plants were analysed in further experiments. First, we examined the survival rate of these lines after heat stress. Briefly, 7-day-old seedlings grown at 22 °C were subjected to heat-stress treatment at 45 °C for 2 h. After recovery at 22 °C for 7 days, only 10% of *fer1-2-3-4* and *fer1-3-4* plants survived, whereas approximately 70% of WT plants survived (Fig. 4a). As shown in Fig. 4b, *TaFER-5B* overexpression functionally complemented the heat stress-sensitive phenotype of *fer1-2-3-4* plants. In addition, *TaFER-5B* transgenic lines also exhibited an enhanced thermotolerance phenotype compared with WT plants (data not show). We further evaluated electrolyte leakage with detached leaves under heat-stress conditions. Detached leaves of *fer1-2-3-4* and *fer1-3-4* leaked more electrolytes than WT and A-CL1 leaves, whereas A-L1 and A-L2 leaked fewer electrolytes than WT leaves (Fig. 4c). Fv/Fm values were A-L2 > A-L1 > A-CL1 > WT > fer1-3-4 > fer1-2-3-4 under heat-stress conditions, whereas no significant differences were observed under control conditions (Fig. 4d). These results also suggest a role of *TaFER-5B* in thermotolerance in *Arabidopsis*.

### Ferritin enhances thermotolerance associated with the ROS scavenging

A number of recent studies have suggested that ferritin protects plant cells from oxidative damage induced by a wide range of stress. Under normal conditions, the *fer1-3-4* mutant leads to enhanced ROS production and increased activity of several reactive oxygen species (ROS) detoxifying enzymes in leaves and flowers [19]. These results indicate that *fer1-3-4* compensates for and bypasses the lack of safe iron storage in ferritins by increasing the capacity of ROS-detoxifying mechanisms [19]. However, when *Arabidopsis* plants are irrigated with 2 mM Fe-EDDHA, the lack of ferritins in *fer1-3-4* plants strongly impairs plant growth and fertility. Thus, under high-iron conditions, free-iron-associated ROS production overwhelms the scavenging mechanisms activated in the *fer1-3-4* mutant [19]. To further determine whether ferritin enhances thermotolerance associated with protecting cells against ROS, we evaluated the accumulation of superoxide radical anions (O^2−^) and H_2_O_2_ under heat-stress conditions. O^2−^ was detected with nitroblue tetrazolium (NBT) staining, and H_2_O_2_ was measured by diaminobenzidine tetrahydrochloride (DAB) staining [25]. We also examined the H_2_O_2_ content and the enzyme activities of catalase (CAT) and glutathione reductase (GR) under normal and heat-stress conditions.

**Fig. 5:**
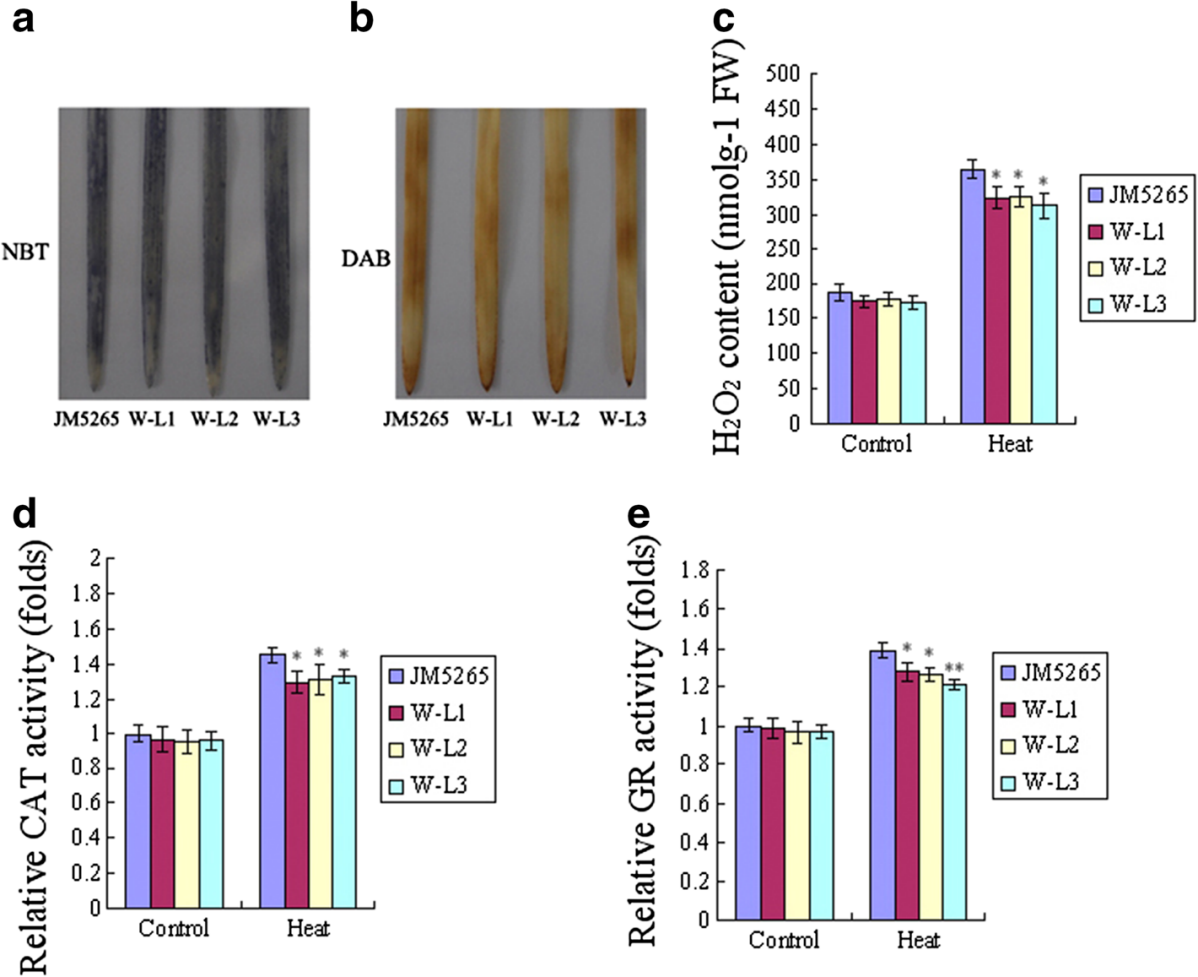
Detection of reactive oxygen species (ROS) in wheat after heat treatment at 45 °C for 1 h. **a** O_2_^−^ accumulation in 7-day-old wheat leaves detected with NBT. **b** H_2_O_2_ accumulation in 7-day-old wheat leaves detected with DAB. **c** H_2_O_2_ content in 7-day-old JM5265 and transgenic wheat seedlings. **d** The activity of the antioxidant enzyme CAT in 7-day-old JM5265 and transgenic wheat seedlings. **e** The activity of the antioxidant enzyme GR in 7-day-old JM5265 and transgenic wheat seedlings. The data represent mean values ± SD of three independent experiments. (* indicates significance at *P* < 0.05; ** indicates significance at *P* < 0.01)

In wheat, transgenic lines accumulated less ROS than JM5265 under stress conditions (Fig. 5a and b). CAT and GR enzyme activities were also positively correlated with ROS content (Fig. 5c and d). These results indicate that overexpression of *TaFER-5B* in wheat effectively alleviates the accumulation of ROS.

**Fig. 6:**
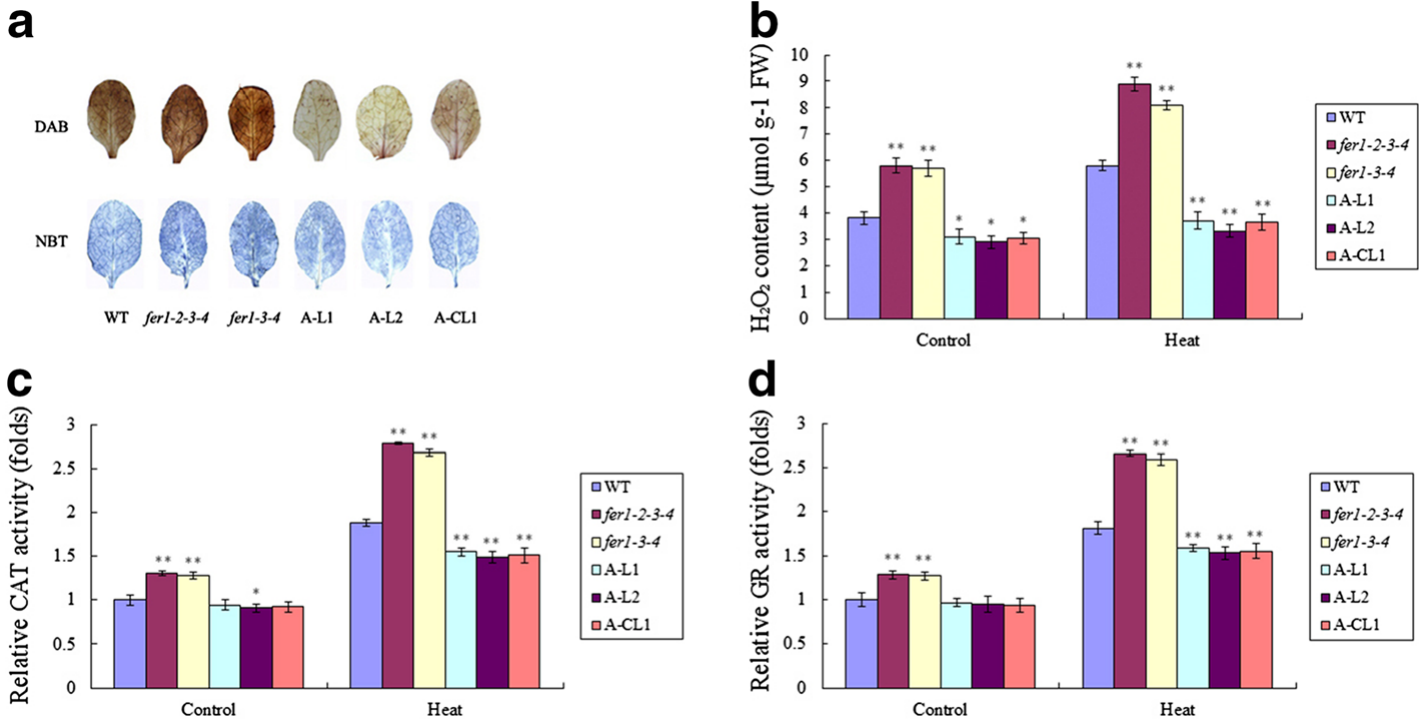
Detection of reactive oxygen species (ROS) in *Arabidopsis*. **a** H_2_O_2_ accumulation was detected with DAB in 3-week-old rosette leaves after heat treatment at 45 °C for 1 h. O_2_^−^ accumulation was detected with NBT in 3-week-old rosette leaves after heat treatment at 45 °C for 1 h. **b** H_2_O_2_ content in 10-day-old seedlings after heat treatment at 45 °C for 1 h. **c** The activity of the antioxidant enzyme CAT in 10-day-old seedlings after heat treatment at 45 °C for 1 h. **d** The activity of the antioxidant enzyme GR in 10-day-old seedlings after heat treatment at 45 °C for 1 h. The data represent mean values ± SD of three independent experiments. (* indicates significance at *P* < 0.05; ** indicates significance at *P* < 0.01)

In *Arabidopsis*, even under normal conditions, differences were noted among *fer1-2-3-4, fer1-3-4, A-L1, A-L2, A-CL1* and WT plants. Compared with WT, *fer1-2-3-4* and *fer1-3-4* exhibited enhanced H_2_O_2_ content and CAT and GR activities, consistent with a previous report [19]. In overexpression and complemented lines, the O^2−^ and H_2_O_2_ content and the two enzyme activities decreased (Fig. 6a, b, c and d). These results indicate that overexpression of *TaFER-5B* in *Arabidopsis* effectively alleviated the accumulation of ROS.

### *TaFER-5B* overexpression also enhances tolerance to drought stress, oxidative stress and excess iron stress associated with the ROS scavenging

**Fig. 7:**
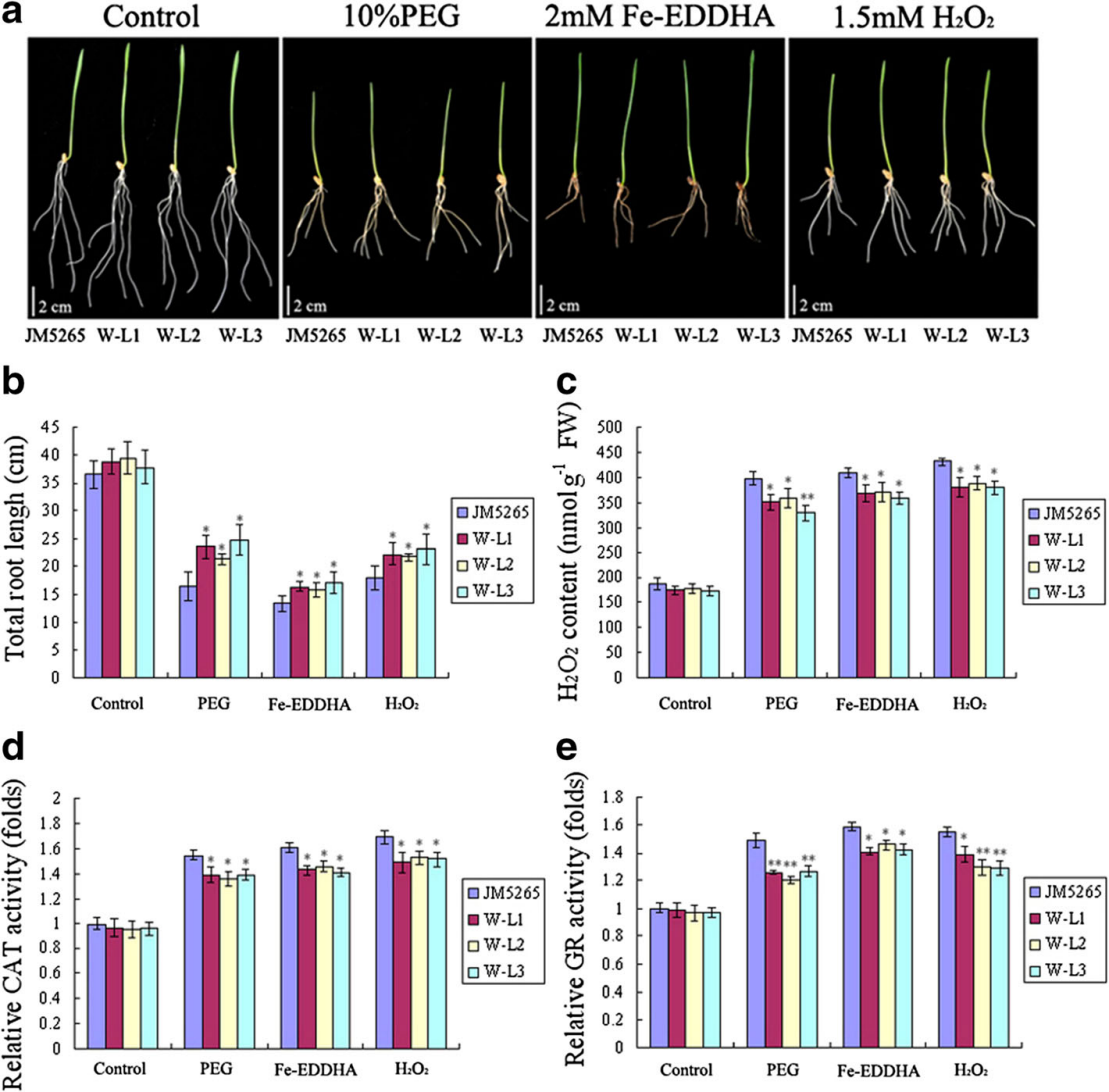
Drought, oxidative, excess iron stress tolerance assay and ROS accumulation analysis of *TaFER-5B* transgenic wheat plants. **a** Phenotypes of 10-day-old wheat plants overexpressing *TaFER-5B* under control, mannitol, Fe-EDTA and H_2_O_2_ conditions. **b** Total root length statistics for the roots of the 10-day-old seedlings in (**a**). **c** H_2_O_2_ content in 10-day-old seedlings in (**a**). **d** The activity of the antioxidant enzyme CAT in the seedlings in (**a**). **e** The activity of the antioxidant enzyme GR in the seedlings in (**a**). The data represent mean values ± SD of three independent experiments. (* indicates significance at *P* < 0.05; ** indicates significance at *P* < 0.01)

**Fig. 8:**
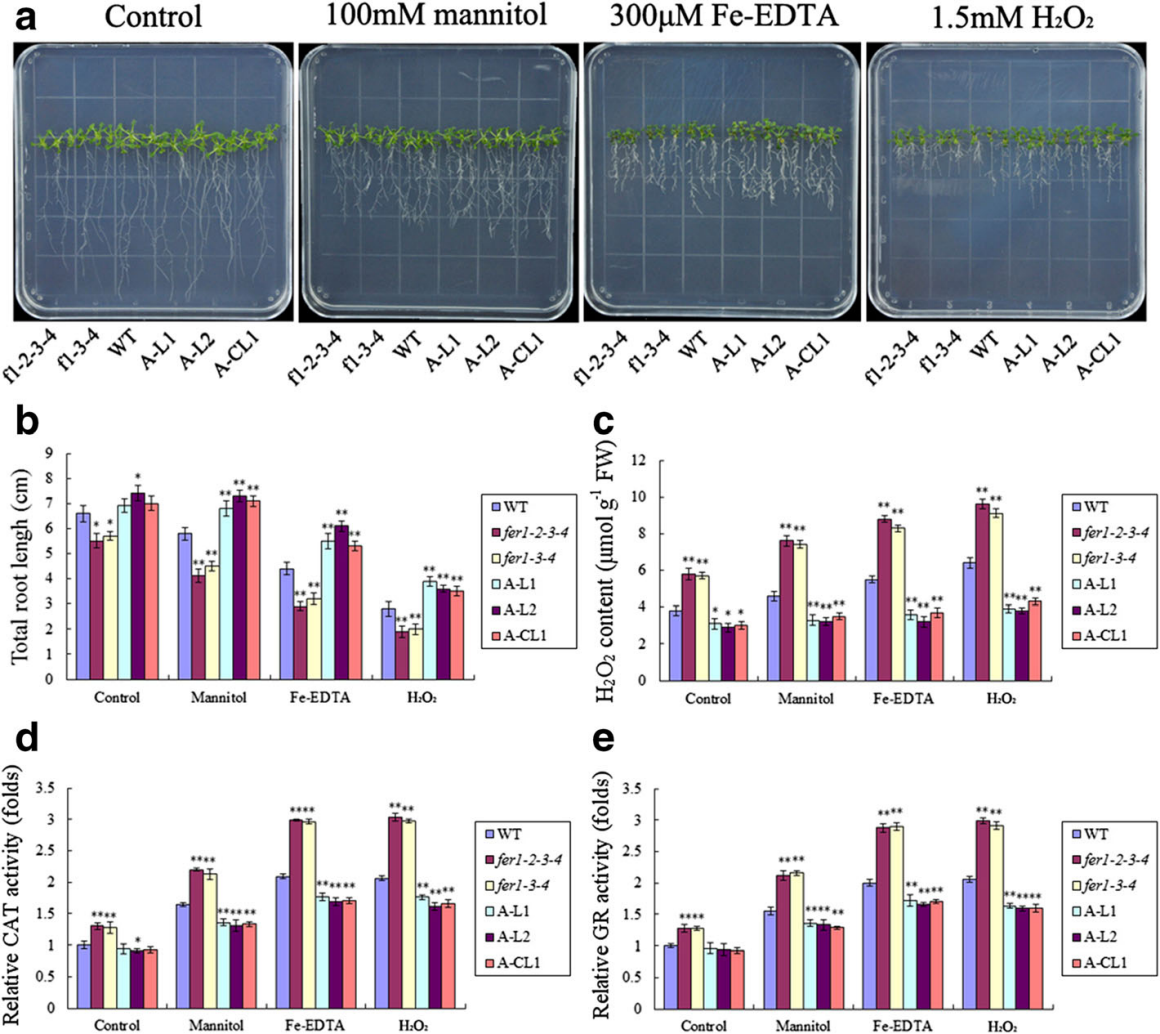
Drought, oxidative, excess iron stress tolerance assay and ROS accumulation analysis of *Arabidopsis* ferritin-lacking mutants, *TaFER-5B*-overexpressing lines and complemented lines. **a** Phenotypes of 10-day-old *Arabidopsis* ferritin-lacking mutants, *TaFER-5*B-overexpressing lines and complemented lines before and after mannitol, Fe-EDTA and H_2_O_2_ treatment. **b** Total root length statistics for the roots of the 10-day-old seedlings in (**a**). **c** H_2_O_2_ content in the 10-day-old seedlings in (**a**). **d** The activity of the antioxidant enzyme CAT in the seedlings in (**a**). **e** The activity of the antioxidant enzyme GR in the seedlings in (**a**). The data represent mean values ± SD of three independent experiments. (* indicates significance at *P* < 0.05; ** indicates significance at *P* < 0.01)

As mentioned above, *TaFER-5B* was also induced by PEG, H_2_O_2_ and excess iron treatment (Fig. 2b, c and d), suggesting that *TaFER-5B* may be involved in an intricate network for abiotic stress responses. To investigate the role of *TaFER-5B* in these abiotic stresses, we examined the effect of *TaFER-5B* on drought stress, oxidative stress and excess iron stress tolerance in wheat. The *TaFER-5B* transgenic lines exhibited significantly greater total root length in the presence of 10% PEG, 2 mM Fe-EDDHA or 1.5 mM H_2_O_2_ (Figs. 7a and b). The H_2_O_2_ content and CAT and GR enzyme activities were also dramatically decreased in the *TaFER-5B* transgenic lines (Fig. 7c, d and e). These results suggest that overexpression of *TaFER-5B* in wheat enhances drought, oxidative and excess iron stress tolerance associated with the ROS scavenging. In *Arabidopsis*, we also investigated tolerance to the above stresses in *fer1-2-3-4, fer1-3-4, A-L1, A-L2, A-CL1* and WT plants. After 10 d of exposure to 10% PEG, 2 mM Fe-EDDHA or 1.5 mM H_2_O_2_, the total roots of the A-L1, A-L2 and A-CL1 lines were significantly longer than those of WT and *fer1-2-3-4* (Fig. 8a and b). Consistent with this result, H_2_O_2_ content and CAT and GR activities were significantly decreased in the A-L1, A-L2 and A-CL1 lines compared to WT and *fer1-2-3-4* (Fig. 8c, d and e). These data indicate that *TaFER-5B* is essential for enhancing abiotic stress tolerance associated with the ROS scavenging.

### *TaFER-5B* overexpression improves the iron content in the leaves but not seeds of transgenic plants

**Fig. 9:**
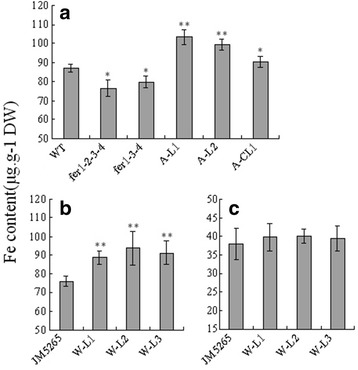
Iron content in *Arabidopsis* and wheat. **a** Iron content in leaves of 3-week-old *TaFER-5B* transgenic plants, ferritin-lacking *Arabidopsi*s mutants and complemented lines in *Arabidopsis* before bolting. **b** Iron content in wheat shoots of 10-day-old *TaFER-5B* transgenic plants. **c** Iron content in dry wheat seeds of *TaFER-5B* transgenic plants. The data represent mean values ± SD of three independent experiments. (* indicates significance at *P* < 0.05; ** indicates significance at *P* < 0.01)

To confirm the function of *TaFER-5B* in improving iron content in transgenic plants, ICP-AAS was used to analyse the iron concentration. In the shoots of transgenic *Arabidopsis* plants and mutants before bolting, the iron content displayed the same trend as ferritin protein levels (Fig. 9a and Additional file 6: Figure S4), and 10-day-old transgenic wheat plants exhibited similar results (Fig. 9b). As a major crop, we were more concerned about the iron content in the seeds of transgenic wheat. However, the results revealed no significantly differences in iron content in seeds between JM5265 and transgenic plants (Fig. 9c). This finding is consistent with the previous results [26]. Previous overexpression of *GmFer* in wheat and rice driven by the maize ubiquitin promoter resulted in improvements in the iron content in the vegetable tissue without significant changes in seeds. Supporting this finding, *Arabidopsis* ferritins store only approximately 5% of the total seed iron and do not constitute the major seed iron pool [19].

## Discussion

Heat and drought stress have an adverse impact on crop productivity and quality worldwide. Plants have evolved various response mechanisms for heat and drought stress, particularly molecular responses, to maintain normal life activities [3, 4, 27]. Forward and reverse genetics have been applied to identify key molecular factors that facilitate crop acclimation to environmental stress. In this study, we successfully cloned the gene *TaFER-5B* and elucidated its function in tolerance to heat and other abiotic stresses in *Arabidopsis* and wheat.

### *TaFER-5B* possesses the typical features of plant ferritins

Plant and animal ferritins evolved from a common ancestor gene. Animal ferritins contain two types of subunits, referred to as H- and L-chains. All plant ferritins all share higher identity with the H-chains of animal ferritins. In cereals, there are two ferritin genes per haploid genome. In hexaploid wheat, *TaFer1* and *TaFer2* are located on chromosomes 5 and 4, respectively, and three homeoalleles of each gene are located in the A, B and D genomes, respectively [13]. Similar to other plant ferritin genes, the gene structure of *TaFER-5B* contains seven introns and eight exons (data not show). Ferritin subunits are synthesized as a precursor, and the N-terminal sequence consists of two domains: the transit peptide and the extension peptide (Fig. 1a). The transit peptide domain has higher variability and is absent in the mature ferritin subunit; the transit peptide domain is responsible for plastid localization. The adjacent extension peptide domain present in the mature ferritin subunit is involved in protein stability [28]. In sea lettuce ferritins, the extension peptide contributes to shell stability and surface hydrophobicity [29]. The removal of the extension peptide in pea seed ferritin both increases protein stability and promotes the reversible dissociation of the mature ferritin protein [30]. The secondary structure of pea seed ferritin is highly similar to that of mammalian ferritin [31]. The ferritin cage structure is assembled from 24 individual four-helix bundle subunits (A, B, C, and D in Fig. 1a) and is conserved in plant and animal ferritins. In plants, which diverged from their animal counterparts, the ferritins also contain a smaller and highly conserved C-terminal E-helix that participates in the formation of the fourfold axis and is involved in electron transfer [21].

### The expression of ferritins is modulated by heat stress and other abiotic stresses

In this study, the probe corresponding to *TaFER-5B* was induced by heat stress [14]. RT-qPCR analysis demonstrated that this gene was induced by heat treatment and peaked at 3 h of treatment (Fig. 2a). We also analysed the expression profiles of *TaFER-4A*, *TaFER-4B, TaFER-4D*, *TaFER-5A*, *TaFER-5B* and *TaFER-5D* under drought stress, heat stress and their combination in the expression database (http://wheat.pw.usda.gov/WheatExp/) [32]. *TaFER-5A*, *TaFER-5B* and *TaFER-5D* were all induced by drought stress, heat stress and their combination (Additional file 7: Figure S5). These results indicate that, in addition to functioning as the iron storage protein in the development stages, plant ferritins may also function as stress-responsive proteins. Previous studies have demonstrated that ferritin gene expression is induced by heat treatment. *PpFer4* expression was significantly induced by 6 h of treatment at 40 °C [33]. In barley caryopses, heat treatment at 0.5 h, 3 h and 6 h could induced the expression of the ferritin gene-corresponding probe Barley1_02716 [34]. In cotton leaves, the expression of the ferritin gene-corresponding probe Gra.2040.1.A1_s_at was induced in cultivar Sicala45 but not in cultivar Sicot53 after 42 °C treatment. We also analysed the expression profiles of the four *Arabidopsis* ferritin genes after heat stress in the expression database (http://jsp.weigelworld.org/expviz/expviz.jsp). After 38 °C treatment, *AtFER1*, *AtFER3* and *AtFER4* expression was induced gradually and peaked at 3 h. After recovery to normal conditions, the expression levels of these three genes gradually decreased to normal levels. *AtFER2* accumulates in dry seed. *AtFER2* abundance in vegetative organs is minimal, and its expression is not induced by heat. Taken together, we believe that induction of the plant ferritin gene by heat is a common phenomenon and that ferritin genes are involved in coping with heat stress.

We analysed the 2000-bp sequence of *TaFER-5B* upstream of the start codon and did not identify the typical heat stress responsive element (HSE) (Additional file 8: Figure S6). This result indicates that *TaFER-5B* expression is not regulated by heat transcription factors but by other pathways. We also analysed the promoter region of the four *Arabidopsis* ferritin genes. HSEs were identified in the promoter regions of *AtFER2*, *AtFER3* and *AtFER4* but not in *AtFER1* (Additional file 8: Figure S6). These results indicate that heat transcription factors participate in the regulation of ferritin genes; however, other pathways are also involved because some ferritin genes are induced by heat even though their promoter regions do not have the typical HSE.

Under drought or other stress conditions, free iron in plants increases rapidly and induces the expression of ferritin to cope with the stress [19]. This phenomenon may be a regulatory mechanism to induce the expression of ferritin under heat-stress conditions. In addition, in the *OsHDAC1* overexpression lines, ferritin gene expression is decreased [35]. *AtFER1* and *AtFER2* are the target genes of *AtGCN5*, and the expression levels of *AtFER3* and *AtFER4* are decreased in the mutant *gcn5-1* [36]. These results indicate that histone modification may be involved in the regulation of the ferritin gene.

Some abiotic stress and hormone responsive elements were identified in the promoter sequence of *TaFER-5B* (Additional file 8: Figure S6). The expression of *TaFER-5B* was also induced by PEG, H_2_O_2_ and Fe-EDDHA treatment (Fig. 2b, c, d). We also analysed the expression profiles of the four ferritin genes under abiotic stress conditions using the *Arabidopsis* expression database (http://jsp.weigelworld.org/expviz/expviz.jsp). After cold treatment, only the expression of *AtFER3* was induced. Under salt-stress conditions, the expression of *AtFER1* and *AtFER3* was increased 6-fold and 3-fold, respectively, but the expression of *AtFER4* was not altered. Under drought-stress conditions, only the expression of *AtFER1* and *AtFER3* was induced. In rice, *OsFER2* was induced by Cu, paraquat, SNP (a nitric oxide donor) and iron [37]. Ferritin is one of the genes up-regulated in response to drought in the SSH cDNA library of soybean nodules [16]. These results indicated that ferritin gene expression is induced by various abiotic stress treatments. Thus, the regulation of the ferritin gene is very complicated.

### Ferritin plays an important role in the defence against heat and other abiotic stresses in plants

In this study, we demonstrated that *TaFER-5B* is induced by heat stress and other abiotic stresses. Overexpression of *TaFER-5B* in both wheat and *Arabidopsis* enhanced heat, drought, oxidative and excess iron stress tolerance compared with control plants. Transgenic tobacco plants ectopically expressing *MsFer* are more tolerant to oxidative damage and pathogens compared with WT plants [38]. Transgenic grapevine plants overexpressing *MsFer* were used to evaluate the tolerance to oxidative and salt stress [17]. What is the mechanism by which plant ferritin improves tolerance to abiotic stress? We evaluated the accumulation of O^2−^ and H_2_O_2_ in transgenic plants and control under heat stress, which revealed that the transgenic plants accumulated less O^2−^ and H_2_O_2_. High temperature induces the production of ROS and cause oxidative stress. We hypothesized that when a plant is under oxidative stress caused by high temperature, ferritin transforms toxic Fe^2+^ to the non-toxic chelate complex and protect cells against oxidative stress. When no additional ROS scavenging mechanisms are available, the function of ferritin is amplified and plays an important role.

## Conclusions

Ferritins are conserved throughout the plant kingdom, and two genes per genome have been identified in all studied cereals. In this study, we cloned *TaFER-5B* from wheat and determined that *TaFER-5B* is induced by heat stress and other abiotic stresses. The relationship between *TaFER-5B* and abiotic stress tolerance was characterized. Our results suggest that *TaFER-5B* plays an important role in enhancing tolerance to heat stress and other abiotic stresses associated with the ROS scavenging.

## Methods

### Plant materials, growth conditions, and stress treatments

The common wheat genotype “TAM107” [39], which has a thermotolerant phenotype released by Texas A&M University in 1984, was used in this study. Seeds were surface-sterilized and soaked overnight in the dark at room temperature. The sprouted seeds were transferred to petri dishes with filter paper and cultured in water (25 seedlings per dish). The seedlings were grown in a growth chamber at a temperature, light cycle and humidity of 22 °C/18 °C (day/night), 12 h/12 h (light/dark), and 60%, respectively. Briefly, 10-day-old wheat seedlings were treated. Drought stress, oxidative stress and excess iron stress were applied by replacing water with PEG-6000 (20%), H_2_O_2_ (5 mM) or Fe-EDDHA (10 mM), respectively. For high-temperature treatments, seedlings were transferred to a growth chamber maintained at 40 °C. Untreated control seedlings were grown in the growth chamber under normal conditions. Leaves were collected from the seedlings at 1 h, 3 h, 6 h and 12 h after stress treatment, frozen immediately in liquid nitrogen and stored at -80 °C until RNA isolation and other analyses.

*Arabidopsis thaliana* ecotype Col-0 was used as WT. The T-DNA insertion lines *fer1-1* (SALK_055487), *fer2-1 (*SALK_002947*), fer3-1* (GABI-KAT_496A08) and *fer4-1* (SALK_068629) were obtained from The *Arabidopsis* Information Resource (TAIR, http://arabidopsis.org) as described previously [19, 40]. *fer1-3-4* and *fer1-2-3-4* were generated as described in previous studies [19, 24]. Seeds were surface-sterilized and cold treated at 4 °C for 3 days in the dark, and then seedlings were grown at 22 °C on horizontal plates containing Murashige and Skoog (MS) medium (pH 5.8) solidified with 0.8% agar unless otherwise specified. Plants were grown at 22 °C under a 16 h*/*8 h (light/dark) photoperiod in the greenhouse.

### Cloning of the *TaFER* gene and sequence analysis

Total RNA was extracted with TRIzol reagent (Invitrogen), and purified RNA was treated with DNase I. Subsequently, 2 μg of total RNA was reverse transcribed by M-MLV reverse transcriptase (Promega, USA). Based on the candidate probe sequence (Ta.681.1.S1_x_at), a pair of gene-specific primers was used to amplify *TaFER*. The primer sequences are listed in Additional file 9: Table S1 (1, 2).

Database searches of the nucleotide and deduced amino acid sequences were performed by NCBI/GenBank/Blast. Sequence alignment and similarity comparisons were performed using DNAMAN. Sequence alignments were performed by ClustalX, and the neighbour-joining tree was constructed using the MEGA5.1 program.

### Expression pattern analysis of the *TaFER-5B* gene in wheat

Quantitative real-time PCR (RT-qPCR) was performed to determine the relative expression pattern of *TaFER-5B* with specific primers designed previously [13]. The 2^-ΔΔC^_T_ method [41] was used to quantify the relative expression levels of *TaFER-5B*, and wheat *β-actin* was used as the endogenous control. Each experiment was independently repeated three times. Additional file 9: Table S1 (3, 4, 9, 10) lists the RT-qPCR primers.

### Immunoblot analysis

Total protein extracts were prepared with sample buffer (250 mM Tris–HCl, pH 6.8, 10% SDS, 0.5% bromophenol blue, 50% glycerol and 5% β-mercaptoethanol). Protein concentration was measured with a Coomassie Brilliant Blue binding assay. Protein samples were separated by SDS-PAGE and blotted onto PVDF membranes for immunoblot analysis. Immunodetection of ferritin was performed using rabbit anti-FER polyclonal antibody (Agrisera).

### Generation of transgenic wheat lines overexpressing *TaFER-5B*

The complete ORF of *TaFER-5B* driven by the maize ubiquitin promoter was inserted into vector pBract806. The resulting expression constructs were used for the production of *TaFER-5B*-overexpressing transgenic wheat lines.

Immature embryos from cultivar JM5265 were used for wheat transformation via the particle bombardment method. The presence of a *TaFER-5B* transgene in the transgenic lines was verified by PCR. Additional file 9: Table S1 (5, 6) lists the primers used for PCR.

### Thermotolerance assay in wheat

Seeds of JM5265 obtained from Institute of Cereal and Oil Crops of the Hebei Academy of Agriculture and Forestry Sciences and transgenic lines were sown in pots containing potting soil and grown under the above-mentioned conditions. 5-day-old seedlings were directly exposed to 45 °C for 18 h, typically at 9 AM, in a lighted growth chamber and then shifted to 22 °C to the previous day/night cycle for recovery. The results were photographically documented after 5-d at 22 °C.

### Transgenic *Arabidopsis* plant generation

The complete ORF of *TaFER-5B* was amplified with primers (Additional file 9: Table S1). The PCR product was digested with *Xba* I and *Kpn* I and cloned into the pCAMBIA1300 vector (driven by the CaMV 35S promoter). *Agrobacterium tumefaciens* strain GV3101 containing this binary construct was used to transform *Arabidopsis* plants. Transformants were selected on MS medium containing hygromycin (30 mg/L) and subject to PCR amplification.

### Thermotolerance assay in *Arabidopsis*

Seeds of WT and transgenic lines were sown on a petri dish with approximately 33 mL of solid medium and grown under the above-mentioned conditions. The plated 7-day-old seedlings were directly exposed to 45 °C for 120 min, typically at 9 AM, in an illuminated growth chamber and then shifted to 22 °C to the previous day/night cycle for recovery. The results were photographically documented after 5 to 7 days at 22 °C. The survival rate was the ratio of surviving seedlings to total seedlings planted. Seedlings that were still green and producing new leaves were scored as surviving seedlings.

### Ion leakage assay

Electrolyte leakage was measured as previously described [42]. Leaf segments of uniform maturity were cut into discs and washed three times with de-ionized water to eliminate external residues. Six discs were placed in test tube flasks with 20 mL of de-ionized water and incubated at 42 °C for 1 h. After incubation at room temperature for 24 h, the conductivity of the solution was read with a Horiba Twin Cond B-173 conductivity metre (HORIBA Ltd, Kyoto, Japan) and noted as T1. Then, the sample was boiled for 15 min to kill the tissues, followed by incubation at room temperature for 24 h. Then, the conductivity of this solution was recorded as T2. Ion permeability was measured as T1/T2. The experiment was independently repeated in triplicate.

### Chlorophyll fluorescence measurement

The maximum efficiency of photosystem II (PSII) photochemistry, the Fv/Fm ratio, was measured using a pulse-modulated fluorometer (MINI-PAM, Heinz Walz, Effeltrich, Germany). Heat treatment was performed by transferring the plants to another growth chamber at 38 °C for 2 h. The Fv/Fm ratio was measured immediately after heat stress.

### Determination of H_2_O_2_ content and antioxidant enzyme activities

Plants were harvested for assays of ROS accumulation and antioxidant enzyme activities. H_2_O_2_ content was determined following the protocol of the H_2_O_2_ Colorimetric Assay Kit (Beyotime). The activities of the antioxidant enzymes CAT and GR were determined using the CAT Assay Kit (S0051; Beyotime) and GR Assay Kit (S0055; Beyotime), respectively, according to the manufacturer’s instructions.

### Iron content measurement

Fe contents were analysed by inductively coupled plasma atomic absorption spectrometry (ICP-AAS). Samples were mineralized as described previously [19].

## Additional files

Additional file 1: Coding sequence of *TaFER-5B*. (DOCX 15 kb)
Additional file 2: Figure S1. Phylogenetic tree analysis of plant *FERs* from wheat, *Arabidopsis*, maize, rice and barley. *TaFER-5A*: accession number FJ225137; *TaFER-5B*: accession number FJ225141 and KX025176; *TaFER-5D*: accession number FJ225144; *TaFER-4A*: accession number TC373825; *TaFER-4B*: accession number FJ2251491; *TaFER-4D*: accession number FJ225146; *AtFER1*: accession number AED90364.1 (AT5G01600); *AtFER2*: accession number AEE74997.1 (AT3G11050); *AtFER3*: accession number AEE79476.1 (AT3G56090); *AtFER4*: accession number AEC09810.1 (AT2G40300); *ZmFER1*: accession number X83076.1; *ZmFER2*: accession number X83077.1; *OsFER1*: accession number AK059354.1; *OsFER1*: accession number AK102242.1; *HvFER1*: accession number EF440353; *HvFER2*: accession number AK251285. (JPG 55 kb)
Additional file 3: Phylogenetic data of Additional file 2: Figure S1. (DOCX 16 kb)
Additional file 4: Figure S2.
*TaFER-5B* overexpression in wheat. (A) Confirmation of *TaFER-5B* insertion in JM5265 by PCR analysis of JM5265, PC, W-L1, W-L2 and W-L3 transgenic plants. PC: *Ubi::TaFER-5B* vector was used as the positive control. (B) Transcript levels of *TaFER-5B* as determined by RT-qPCR. *β-actin* was used as the internal control. The data are presented as the mean ± SD of three independent biological replicates. (C) *TaFER-5B* insertion into JM5265 as analysed by Western blot in JM5265, W-L1, W-L2 and W-L3 transgenic plants. (JPG 81 kb)
Additional file 5: Figure S3. Genotyping of WT, *fer1*, *fer2*, *fer3*, *fer4*, *fer1-3-4* and *fer1-2-3-4*. (JPG 232 kb)
Additional file 6: Figure S4. Western blot analysis of the WT and transgenic lines. (A) Western blot of the WT and transgenic plants overexpressing *TaFER-5B*. (B) Western blot of the ferritin-lacking mutants, WT and the complemented lines A-CL1. Rubisco was used as the loading control. (JPG 127 kb)
Additional file 7: Figure S5. Expression analysis of *TaFER4* and *TaFER5* homeologous genes in the RNA-seq expression database WheatExp (http://wheat.pw.usda.gov/WheatExp). (JPG 187 kb)
Additional file 8: Figure S6. Cis-acting regulatory elements predicted in the promoters of *TaFER-5B* from IWGSC database and *AtFER1-4*. Distances shown in base pairs are relative to the start codon (+1). (JPG 174 kb)
Additional file 9: Table S1. Primers used in this paper. (DOCX 15 kb)

## Abbreviations

ABA: Abscisic acid
CAT: Catalase
CS: Chinese spring
DAB: Diaminobenzidine tetrahydrochloride
Fe-EDDHA: Fe-ethylenediaminedi (o-hydroxyphenylacetic) acid
Fv/Fm: The ratio of variable to maximal fluorescence
GR: Glutathione reductase
HSE: Heat stress responsive element
ICP-AAS: Inductively coupled plasma atomic absorption spectrometry
JM5265: Jimai5265
MS: Murashige and Skoog
NBT: Nitroblue tetrazolium
O^2−^: Superoxide radical anions
PCD: Programmed cell death
PEG: Polyethylene glycol
PSII: Photosystem II
ROS: Reactive oxygen species
RT-qPCR: Quantitative real-time PCR
SSH: Suppression subtractive hybridisation
WT: Wild type
