# Supplementary material for: Overexpression of wheat ferritin gene TaFER-5B enhances tolerance to heat stress and other abiotic stresses associated with the ROS scavenging
Source: BMC Plant Biol. 2017 Jan 14;17:14. doi: 10.1186/s12870-016-0958-2 (PMC5237568; doi:10.1186/s12870-016-0958-2)
Supplement: Additional file 1: — Coding sequence of TaFER-5B. (DOCX 15 kb) [file 12870_2016_958_MOESM1_ESM.docx]

**Additional file 1:** Coding sequence of TaFER-5B.

1 ATGTTGCCTA GGGTTGCGCC GTCTCCGGCC ACCGCCGCCG CCGCCGCAGC GGTTGGCCAG

61 CTCTCCGGGG CGGGGCTCGC CGCCGGTTCG GTGAGGCTGC CGGGGCCCCT GCCGTCTGCG

121 GCGGGGTCGG CGGTCTGCTG CCGGGCCGCG GCGAAGGGGA AGGAGGTGCT CAGCGGCGTG

181 ATGTTCCAGC CGTTCGAGGA GCTCAAGGGG GAGCTCTCCC TCGTGCCGCA GGGCAAGGAC

241 CAGTCGCTCG CCAGGCACAA GTTCGTCGAC GAGTGCGAGG CCGCCCTCAA CGAGCAGATC

301 AATGTGGAGT ACAATGCCTC GTACGCGTAT CACTCCCTCT TCGCCTACTT CGACCGCGAC

361 AACGTTGCTC TCAAGGGATT TGCCAAGTTC TTCAAGGAAT CAAGCGACGA GGAGAGGGGA

421 CACGCCGAGA AGTTAATGGA GTACCAGAAC AAACGTGGAG GGAGGGTGAG GCTCCAGTCA

481 ATTGTCACAC CCTTAACCGA GTTCGACCAT GCTGAGAAAG GCGATGCCCT GTATGCAATG

541 GAGTTGGCTC TAGCTCTTGA AAAGCTGGTG AATGAGAAAC TGCACAACCT GCACAGTGTA

601 GCTACAAGGT GCAATGATCC TCAGCTGACC GACTTTGTTG AGAGTGAATT CCTTCAGGAG

661 CAGGTTGACG CCATCAAGAA GATCTCTGAG TATGTGTCGC AGCTGAGAAG AGTCGGCAAA

721 GGCCACGGAG TGTGGCACTT CGACCAGATG CTGCTTGAGG AGGCAGCTTG A
