# Supplementary figures and images for: Overexpression of wheat ferritin gene TaFER-5B enhances tolerance to heat stress and other abiotic stresses associated with the ROS scavenging
Source: BMC Plant Biol. 2017 Jan 14;17:14. doi: 10.1186/s12870-016-0958-2 (PMC5237568; doi:10.1186/s12870-016-0958-2)

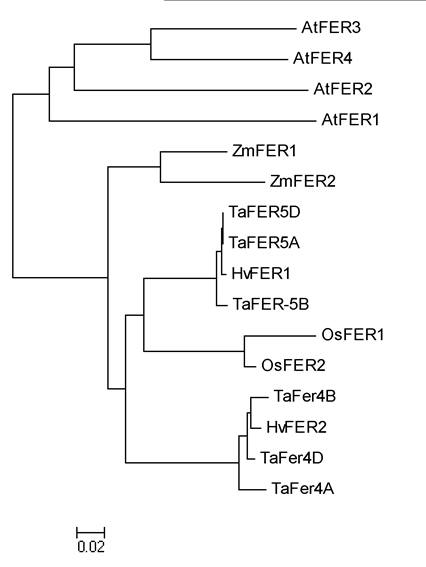

Supplement: Additional file 2: Figure S1. — Phylogenetic tree analysis of plant FERs from wheat, Arabidopsis, maize, rice and barley. TaFER-5A: accession number FJ225137; TaFER-5B: accession number FJ225141 and KX025176; TaFER-5D: accession number FJ225144; TaFER-4A: accession number TC373825; TaFER-4B: accession number FJ2251491; TaFER-4D: accession number FJ225146; AtFER1: accession number AED90364.1 (AT5G01600); AtFER2: accession number AEE74997.1 (AT3G11050); AtFER3: accession number AEE79476.1 (AT3G56090); AtFER4: accession number AEC09810.1 (AT2G40300); ZmFER1: accession number X83076.1; ZmFER2: accession number X83077.1; OsFER1: accession number AK059354.1; OsFER1: accession number AK102242.1; HvFER1: accession number EF440353; HvFER2: accession number AK251285. (JPG 55 kb) [file 12870_2016_958_MOESM2_ESM.jpg]

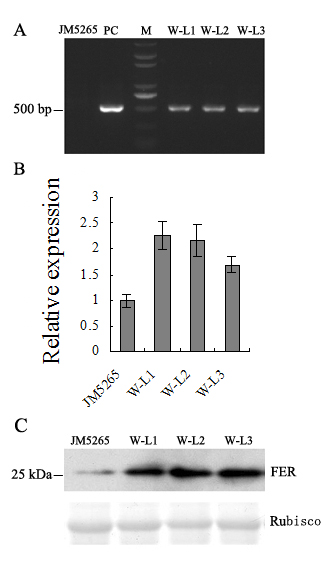

Supplement: Additional file 4: Figure S2. — TaFER-5B overexpression in wheat. (A) Confirmation of TaFER-5B insertion in JM5265 by PCR analysis of JM5265, PC, W-L1, W-L2 and W-L3 transgenic plants. PC: Ubi::TaFER-5B vector was used as the positive control. (B) Transcript levels of TaFER-5B as determined by RT-qPCR. β-actin was used as the internal control. The data are presented as the mean ± SD of three independent biological replicates. (C) TaFER-5B insertion into JM5265 as analysed by Western blot in JM5265, W-L1, W-L2 and W-L3 transgenic plants. (JPG 81 kb) [file 12870_2016_958_MOESM4_ESM.jpg]

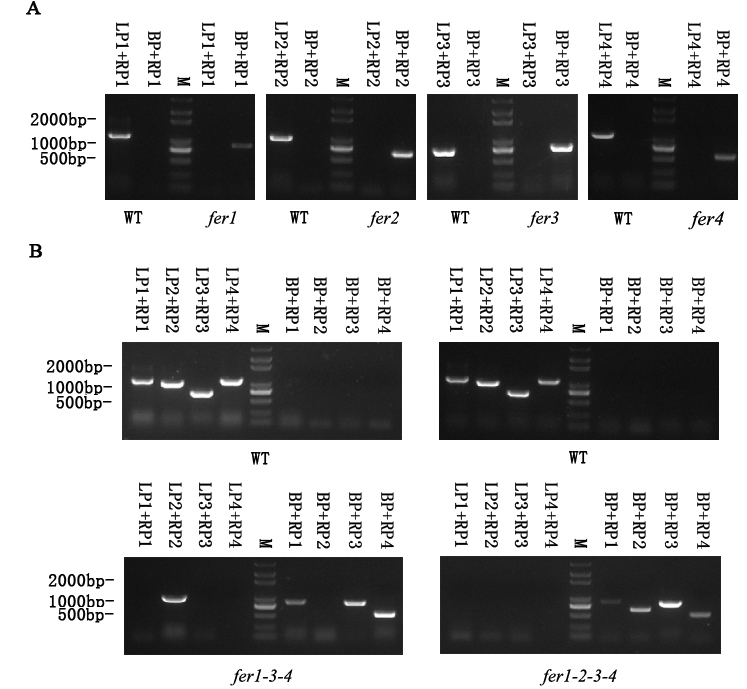

Supplement: Additional file 5: Figure S3. — Genotyping of WT, fer1, fer2, fer3, fer4, fer1-3-4 and fer1-2-3-4. (JPG 232 kb) [file 12870_2016_958_MOESM5_ESM.jpg]

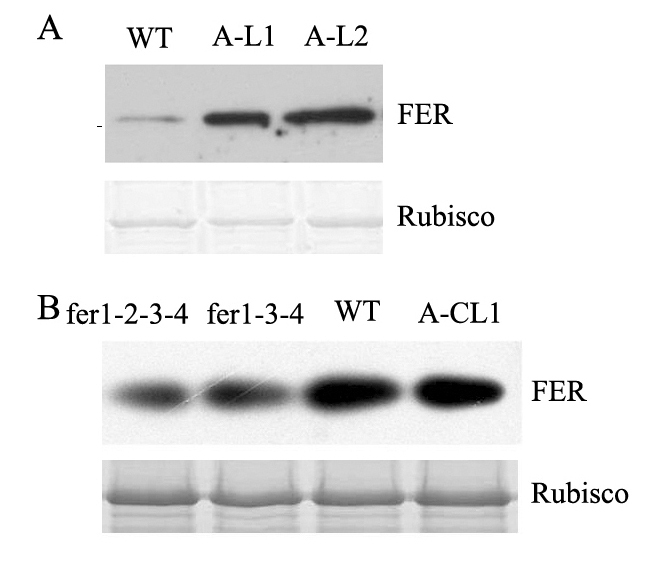

Supplement: Additional file 6: Figure S4. — Western blot analysis of the WT and transgenic lines. (A) Western blot of the WT and transgenic plants overexpressing TaFER-5B. (B) Western blot of the ferritin-lacking mutants, WT and the complemented lines A-CL1. Rubisco was used as the loading control. (JPG 127 kb) [file 12870_2016_958_MOESM6_ESM.jpg]

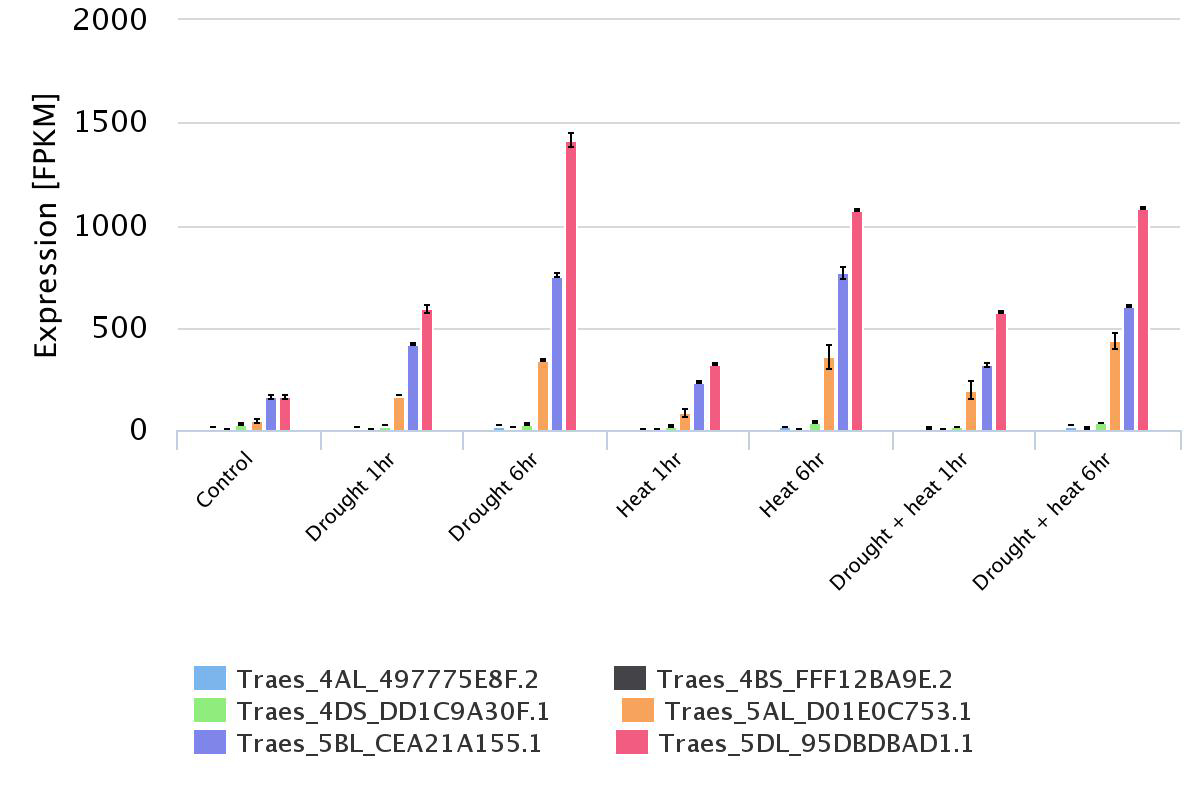

Supplement: Additional file 7: Figure S5. — Expression analysis of TaFER4 and TaFER5 homeologous genes in the RNA-seq expression database WheatExp (http://wheat.pw.usda.gov/WheatExp). (JPG 187 kb) [file 12870_2016_958_MOESM7_ESM.jpg]

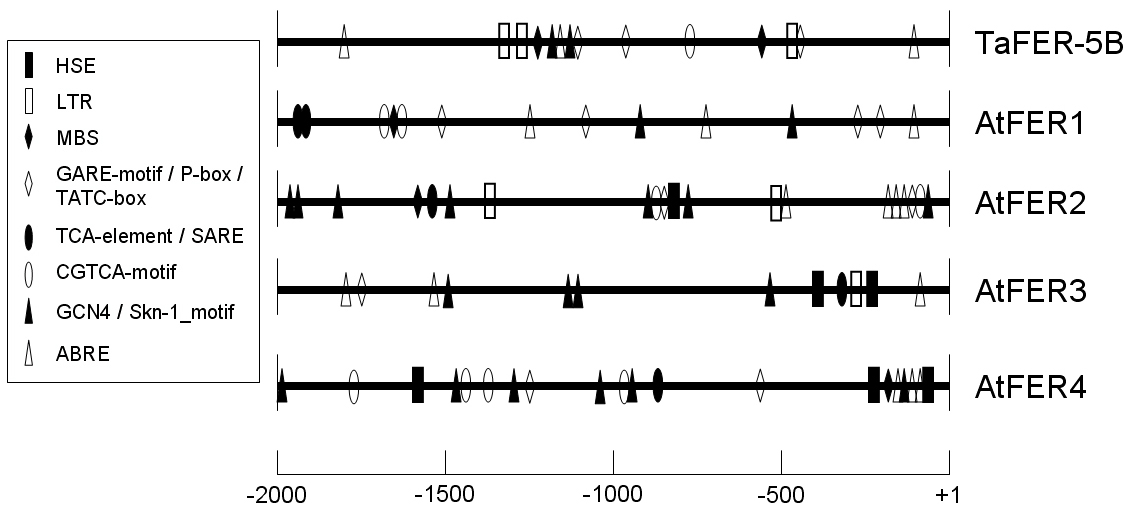

Supplement: Additional file 8: Figure S6. — Cis-acting regulatory elements predicted in the promoters of TaFER-5B from IWGSC database and AtFER1-4. Distances shown in base pairs are relative to the start codon (+1). (JPG 174 kb) [file 12870_2016_958_MOESM8_ESM.jpg]
