# Supplementary material for: Overexpression of wheat ferritin gene TaFER-5B enhances tolerance to heat stress and other abiotic stresses associated with the ROS scavenging
Source: BMC Plant Biol. 2017 Jan 14;17:14. doi: 10.1186/s12870-016-0958-2 (PMC5237568; doi:10.1186/s12870-016-0958-2)
Supplement: Additional file 3: — Phylogenetic data of Additional file 2: Figure S1. (DOCX 16 kb) [file 12870_2016_958_MOESM3_ESM.docx]

**Additional file 3:** Phylogenetic data of Figure S1.

>TaFER-5B FJ225141.1 and KX025176

MLPRVAPSPATAAAAAVGQLSGAGLTAGSVRLPGALPSAAGSAVCCRAAAKGKEVLSGVMFQPFEELKGELSLVPQGKDQSLARHKFVDECEAALNEQINVEYNASYAYHSLFAYFDRDNVALKGFAKFFKESSDEERGHAEKLMEYQNKRGGRVRLQSIVTPLTEFDHPEKGDALYAMELALALEKLVNEKLHNLHSVATRCNDPQLTDFVESEFLQEQVDAIKKISEYVSQLRRVGKGHGVWHFDQMLLEEAA

>TaFER-5A FJ225144.1

MLPRVAPSPATAAAAAAVGQLSGAGLAAGSVRLPGPLPSAAGSAVCCRAAAKGKEVLSGVMFQPFEELKGELSLVPQGKDQSLARHKFVDECEAALNEQINVEYNASYAYHSLFAYFDRDNVALKGFAKFFKESSDEERGHAEKLMEYQNKRGGRVRLQSIVTPLTEFDHAEKGDALYAMELALALEKLVNEKLHNLHSVATRCNDPQLTDFVESEFLQEQVDAIKKISEYVSQLRRVGKGHGVWHFDQMLLEEAA

>TaFER-5D FJ225137.1

MLPRVAPSPATAAAAAVGQLSGAGLTAGSVRLPGALPSAAGSAVCCRAAAKGKEVLSGVMFQPFEELKGELSLVPQGKDQSLARHKFVDECEAALNEQINVEYNASYAYHSLFAYFDRDNVALKGFAKFFKESSDEERGHAEKLMEYQNKRGGRVRLQSIVTPLTEFDHPEKGDALYAMELALALEKLVNEKLHNLHSVATRCNDPQLTDFVESEFLQEQVDAIKKISEYVSQLRRVGKGHGVWHFDQMLL

>TaFER-4A TC373825

MLLRIASSPAAVAAASQLSASSPAHGYARLPPLAKVSSTACRAAGKGKKEEVLLSGVMFQPFEELKGELSLVPQAEGQSLARQKFVDECEAAINEQINVEYNASYAYHSLYAYFDRDNVALKGFAKFFRESSDEEREHAEMLMEYQNRRGGRVRLQSIVTPLTEFDHSEKGDALYAMELALALEKLVNEKLHNLHSVATRCNDPQLSDFVESQFLQEQVVAVKKISEYVTQLRRIGKGHGVWHFDRMLLEEEA

>TaFER-4B FJ225149.1

MFLRIASSPAAVAAASQLSAPGPAHGSARLPPLAKGSSTATACRAAGKGNKEEVLLSGVMFQPFEELKGELSLVPQAEGQSLARQKFVDECEAAINEQINVEYNASYAYHSLYAYFDRDNVALKGFAKFKESSDEEREHAEMLMEYQNRRGGRVRLQSTVTPLTEFDHSEKGDALYAMELALALEKLVNEKLHNLHSVATRCNDPQLSDFVESQFLQEQVDAVKKISEYVTQLRRIGKGHGVWHFDRMLLEEEA

>TaFER-4D FJ225146.1

MLLRIASSPAAVAAASQLSAPGPAHGSARLPPLAKGPSTACRAAGKGKKEEVLLSGVMFQPFEELKGELSLVPQAEGQSLARQKFVDECEAAINEQINVEYNASYAYHSLYAYFDRDNVALKGFAKFFKESSDEEREHAEMLMEYQNRRGGRVRLQSIVTPLTEFDHSEKGDALYAMELALALEKLVNEKLHNLHSVATRCNDPQLSDFVESQFLQEQVDAVKKISEYVTQLRRIGKGHGVWHFDRMLLEEEA

>AtFER1 AED90364.1 (AT5G01600)

MASNALSSFTAANPALSPKPLLPHGSASPSVSLGFSRKVGGGRAVVVAAATVDTNNMPMTGVVFQPFEEVKKADLAIPITSHASLARQRFADASEAVINEQINVEYNVSYVYHSMYAYFDRDNVAMKGLAKFFKESSEEERGHAEKFMEYQNQRGGRVKLHPIVSPISEFEHAEKGDALYAMELALSLEKLTNEKLLNVHKVASENNDPQLADFVESEFLGEQIEAIKKISDYITQLRMIGKGHGVWHFDQMLLN

>AtFER2 AEE74997.1 (AT3G11050)

MLHKASPALSLLSSGYTGGGNLFPPSRNSSNLLFSPSGSRFSVQAAKGTNTKSLTGVVFEPFEEVKKEMELVPTTPFVSLARHKFSDDSESAINDQINVEYNVSYVYHALYAYFDRDNVGLKGFAKFFNDSSLEERGHAEMFMEYQNKRGGRVKLQSILMPVSEFDHEEKGDALHAMELALSLEKLTNEKLLKLQSVGVKNNDVQLVDFVESEFLGEQVEAIKKISEYVAQLRRIGKGHGVWHFDQMLLNDEV

>AtFER3 AEE79476.1 (AT3G56090)

MLLKAASTFSLLNIHGEKKDISPLFSSSSSISSPVSSGKSGNLSFPLRASKSSTTTTSTLSGVVFEPFEEVKKEMDLVPSGQQLSLARHLYSPECEAAVNEQINVEYNVSYVYHALYAYFDRDNVALKGLAKFFKESSVEEREHAELLMEYQNKRGGRVKLQPMVLPQSEFDHPEKGDALYAMELALSLEKLVNEKLLNLHSVASKNDDVQLADFIESVFLNEQVEAIKKISEYVSQLRRLGKGHGTWHFDQELLGAAA

>AtFER4 AEC09810.1 (AT2G40300)

MLLKTVSSSSSSALSLVNFHGVKKDVSPLLPSISSNLRVSSGKSGNLTFSFRASKSSTTDALSGVVFEPFKEVKKELDLVPTSSHLSLARQKYSDECEAAINEQINVEYNVSYVYHAMYAYFDRDNIALKGLAKFFKESSLEEREHAEKLMEYQNKRGGRVKLQSIVMPLSEFEHVDKGDALYGMELALSLEKLVNEKLLNLHSVASKNNDVHLADFIESEFLTEQVEAIKLISEYVAQLRRVGKGHGTWHFNQMLLEG

>OsFER1 AK059354.1

MLPPRVAPAAAAAAPTYLAAAASTPASVWLPVPRGAGPGAVCRAAGKGKEVLSGVVFQPFEELKGELSLVPQAKDQSLARQKFVDECEAAINEQINVEYNASYAYHSLFAYFDRDNVALKGFAKFFKESSDEERDHAEKLIKYQNMRGGRVRLQSIVTPLTEFDHPEKGDALYAMELALALEKLVNEKLHNLHSVASRCNDPQLTDFVESEFLEEQVEAIKKISEYVAQLRRVGKGHGVWHFDQKLLEEEA

>OsFER2 AK102242.1

MLPPRVAPSSLAAAAAAAPTYLAAAASTPASVWLPVPRGAGAVAVCRAAGKGKEVLSGVVFQPFEELKGELSLVPQAKDQSLARQKFVDECEAAISEQINVEFNASYAYHSLFAYFDRDNVALKGFAKFFKESSDEERDHAEKLMKYQNMRGGRVRLQSIVTPLTEFDHPEKGDALYAMELALALEKLVNEKLHNLHSVASRCNDPQLTDFVESEFLEEQVEAIKKISEYVAQLRRVGKGHGVWHFDQKLLEEEA

>ZmFER1 X83076.1

MMLRVSPSPAAAVPTQLSGAPATPAPVVRVAPRGVASPSAGAACRAAGKGKEVLSGVVFQPFEEIKGELALVPQSPDKSLARHKFVDDCEAALNEQINVEYNASYAYHSLFAYFDRDNVALKGFAKFFKESSDEEREHAEKLMEYQNKRGGRVRLQSIVTPLTEFDHPEKGDALYAMELALALEKLVNEKLHNLHGVATRCNDPQLTDFIESDFLEEQVEAINKISKYVAQLRRVGKGHGVWHFDQMLLEEEA

>ZmFER2 X83077.1

MMLRVSSSPAAAVANHLSGGAAATTAPARVTAQRSGVSLSAAAAAGKGKEVLSGVVFQPFEEIKGELALVPQSPDRSLARHKFVDDCEAAINEQINVEYNASYAYHSLFAYFDRDNVALKGFAKFFKESSDEEREHAEKLMEYQNKRGGRVRLQSIVAPLTEFDHPEKGDALYAMELTLALEKLVNEKLHSLHGVATRCNDPQLIDFIESEFLEEQVEAINKVSKYVAQLRRVGNKGHGVWHFDQMLLQEGA

>HvFER1 EF440353

MLPRVAPSPATAAAAVGQLSGAGLTSGSVRLPGALPSAAGSAVCCRAAAKGKEVLSGVMFQPFEELKGELSLVPQGKDQSLARHKFVDECEAALNEQINVEYNASYAYHSLFAYFDRDNVALKGFAKFFKESSDEERGHAEKLMEYQNKRGGRVRLQSIVTPLTEFDHPEKGDALYAMELALALEKLVNEKLHNLHSVATRCNDPQLTDFVESEFLQEQVDAIKKISEYVSQLRRVGKGHGVWHFDQMLLEEAA

>HvFER2 AK251285

MLLRIASSPAAVAAASQLSAPGPAHGSARLPPLAKGPSTACRAARKGNKEEVLLSGVMFQPFEELKGELSLVPQAEGQSLARQKFVDECEAAINEQINVEYNASYAYHSLYAYFDRDNVALKGFAKFFKESSDEEREHAEMLMEYQNRRGGRVRLQSIVTPLTEFDHSEKGDALYAMELALALEKLVNEKLHNLHAVATRCNDPQLSDFVESQFLQEQVDAVKKISEYVTQLRRIGKGHGVWHFDRMLLEEEA
