# Supplementary material for: Overexpression of wheat ferritin gene TaFER-5B enhances tolerance to heat stress and other abiotic stresses associated with the ROS scavenging
Source: BMC Plant Biol. 2017 Jan 14;17:14. doi: 10.1186/s12870-016-0958-2 (PMC5237568; doi:10.1186/s12870-016-0958-2)
Supplement: Additional file 9: Table S1. — Primers used in this paper. (DOCX 15 kb) [file 12870_2016_958_MOESM9_ESM.docx]

**Additional file 9: Table S1.** Primers used in this paper.

| **Number** | **Name** | **Sequence (5′-3′)** |
| --- | --- | --- |
| 1 | *TaFER*-L | ATGTTGCCTAGGGTTGCGCCG |
| 2 | *TaFER*-R | CATCCTCCATTGCCCGTAGTCGT |
| 3 | Q-*FER-5B*-L | GCTTGAAGGACACGGACACG |
| 4 | Q-*FER-5B*-R | TATCACACCTACATCCTCCATTGC |
| 5 | *TaFER-5B*-L2 | GCGTGGACCGTTGCTGCAACT |
| 6 | *TaFER-5B*-R2 | GGGCATCGCCTTTCTCAGCA |
| 7 | *TaFER-5B*-Xba1-L | GC**TCTAGA**ATGTTGCCTAGGGTTGCGCCG |
| 8 | *TaFER-5B*-Kpn1-R | GG**GGTACC**CATCCTCCATTGCCCGTAGTCGT |
| 9 | *β-actin*-L | GGAATCCATGAGACCACCTAC |
| 10 | *β-actin*-R | GACCCAGACAACTCGCAAC |
